# Interpretable intratumoral and peritumoral radiomics using SHAP for predicting microsatellite status in gastric adenocarcinoma: a dual-center study

**DOI:** 10.3389/fonc.2026.1874404

**Published:** 2026-07-20

**Authors:** Jianping Zhu, Peipei He, Haifeng Qiu, Lingling Ying, Jie Chen

**Affiliations:** 1 Department of Radiology, Ningbo Hospital of Integrated Traditional Chinese and Western Medicine, Ningbo, China; 2 Department of Radiology, The People’s Hospital of Pingyang, Wenzhou, China

**Keywords:** gastric cancer, machine learning, microsatellite instability, peritumoral, radiomics

## Abstract

**Objective:**

The purpose of this study was to develop and validate a non-invasive radiomics-based model utilizing contrast-enhanced CT imaging of both intratumoral and peritumoral regions to predict preoperative microsatellite instability (MSI) status in gastric adenocarcinoma (GAC).

**Methods:**

A retrospective cohort comprising 193 patients with histologically confirmed GAC from two separate institutions (Centre 1: n = 115; Centre 2: n = 78) was enrolled. All patients underwent preoperative enhanced CT scans and immunohistochemical assays to determine MSI status. Tumor regions of interest (ROIs), specifically the intratumoral region (IR) and an extended area including the intratumoral and surrounding 3-mm peritumoral regions (IPR), were manually segmented on portal-phase CT images. Radiomics features were extracted from these defined ROIs. Following feature standardization, selection was conducted through inter-observer consistency (ICC), pairwise correlation analyses, and L1-regularized logistic regression. A radiomics signature was subsequently constructed via a support-vector machine (SVM) classifier. Feature contributions were quantified using SHapley Additive exPlanations (SHAP). Independent clinical variables and semantic features derived from CT were employed to develop a separate clinical model. A combined model was then formulated by integrating both radiomics and clinical variables. Receiver operating characteristic (ROC) curves, calibration plots, and decision curve analysis (DCA) were used to evaluate the predictive performance, calibration, and clinical utility of each model.

**Results:**

The combined radiomics-clinical model integrating intratumoral and peritumoral features exhibited superior predictive capability (AUC = 0.891) compared to the standalone clinical model (AUC = 0.771), peritumoral radiomics model (AUC = 0.780), and tumor-clinical integrated model (AUC = 0.784). Calibration curves and decision-curve analysis suggested favorable calibration and potential clinical net benefit of the integrated model.

**Conclusion:**

The integrated model combining clinical variables with radiomic characteristics extracted from the intratumoral plus 3-mm peritumoral region showed promising ability for preoperative MSI prediction in GAC. Because this retrospective study included few MSI-H cases and used IHC as the reference standard, the model should be regarded as a preliminary imaging biomarker that requires prospective multicenter validation before clinical use.

## Introduction

Gastric adenocarcinoma (GAC) remains the fifth most common malignancy and accounts for more than 700,000 deaths annually. Approximately two-thirds of patients present with locally advanced or metastatic disease, for which surgery alone is rarely curative (1). Therefore, perioperative cytotoxic chemotherapy and immune checkpoint inhibitors (ICIs) have become the standard of care (2). MSI–high (MSI-H) tumors constitute 10–15% of GAC, harbor numerous immunogenic frameshift neoantigens, and are heavily infiltrated by activated CD8+ T cells (3). Consequently, PD−1/PD−L1 blockade produces objective responses in approximately 50% of MSI-H cases compared with <15% in microsatellite-stable (MSS) tumors (KEYNOTE−059, −061, −062) (4–6). These findings led the 2023 ASCO and NCCN guidelines to recommend upfront ICI therapy for MSI-H metastatic GAC and universal MSI testing at diagnosis to guide neoadjuvant, adjuvant, and clinical trial decisions (7, 8). Accurate preoperative determination of MSI status could avoid unnecessary cytotoxic therapy, facilitate timely access to ICIs, and reduce morbidity and healthcare costs.

Current MSI determination relies on surgical specimens or endoscopic biopsy, followed by immunohistochemistry (IHC) for MLH1, MSH2, MSH6, and PMS2, PCR fragment analysis, or next−generation sequencing (9). These approaches are invasive, labor−intensive, and susceptible to sampling bias. Endoscopic biopsies sample less than 2% of the tumor volume, frequently miss focal mismatch−repair loss, and are often inadequate in ulcerated, necrotic, or submucosal lesions (10). Postoperative testing may delay treatment decisions, thereby limiting opportunities for individualized neoadjuvant therapy. Therefore, a non−invasive and readily available MSI biomarker is urgently required.

Contrast−enhanced CT, which is routinely performed for staging in newly diagnosed GAC, can be repeated without additional discomfort or cost. Radiomics enables high-throughput extraction of quantitative imaging features describing tumor shape, texture, heterogeneity, and the peritumoral microenvironment, and has been widely explored for tumor staging, early diagnosis, lesion differentiation, prognosis prediction, and treatment-response assessment (11–20). Multimodal imaging, including CT, MRI, PET/CT, and nuclear medicine approaches, may further support personalized oncologic assessment, while PD-1/PD-L1-related biology remains central to immunotherapy decision-making in gastric adenocarcinoma (21–24). In gastric cancer, CT radiomics has been used to predict Lauren histological subtype, HER2 status, tumor mutational burden, and CD8+ tumor-infiltrating lymphocyte density (25, 26). Recently, CT-based MSI signatures in colorectal and gastric cancer have shown encouraging discrimination, particularly when peritumoral regions are incorporated (26–37). Based on these findings, this dual-center study integrated intratumoral and peritumoral CT radiomics with clinical variables to develop a non-invasive preoperative MSI prediction model for GAC, while recognizing that tissue-based MSI testing remains the reference standard.

## Materials and methods

### Patient selection

**Figure 1:**
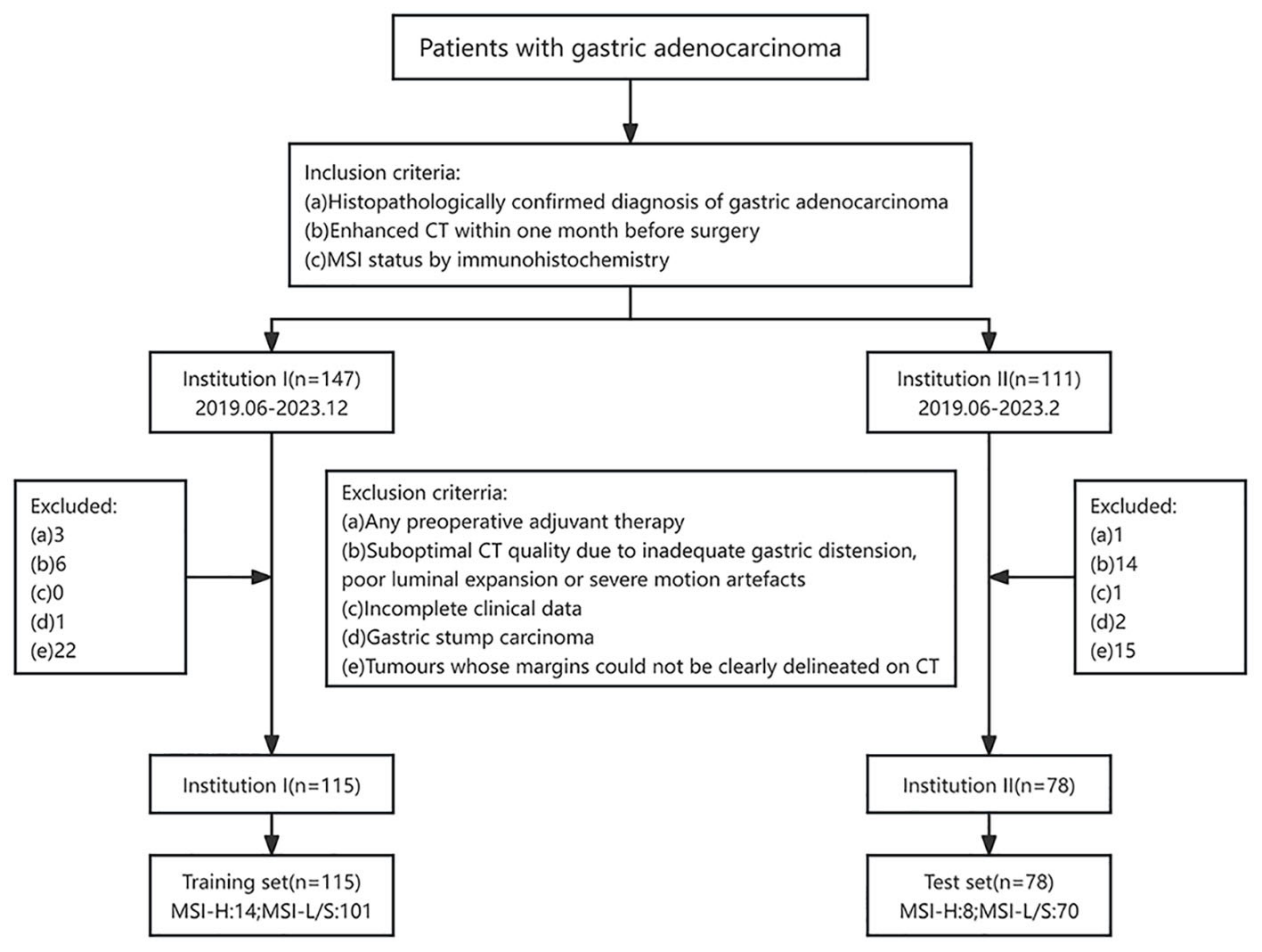
Flowchart of patient selection.

This retrospective two-center study was approved by the institutional review boards of Pingyang People’s Hospital (Institution I) and the Second Ningbo Yinzhou Hospital (Institution II). The requirement for informed consent was waived because of the retrospective design and use of anonymized data. All procedures complied with the Declaration of Helsinki. Between June 2019 and December 2023, 258 consecutive patients with GAC were initially screened. After applying the inclusion criteria (histopathologically confirmed GAC, contrast−enhanced multidetector CT within one month before surgery, and MSI status determined by immunohistochemistry) and the exclusion criteria (any preoperative anticancer therapy; suboptimal CT quality caused by inadequate gastric distension, poor luminal expansion, or severe motion artefacts; incomplete clinical information; gastric stump carcinoma; or tumors with indistinct margins on CT), 193 patients were finally included (**Figure 1**). Patients from Institution I were assigned to the training cohort (n = 115), whereas those from Institution II formed the independent validation cohort (n = 78).

### Data collection

Clinical data from patients’ electronic medical records were retrospectively extracted, encompassing demographic details and relevant clinical characteristics. Semantic CT parameters included clinical T stage (cT), clinical N stage (cN), overall clinical TNM stage (cTNM), Borrmann type, tumor size, and tumor location. Borrmann type was assigned according to conventional macroscopic gastric-cancer morphology, and tumor location was classified as upper, middle, or lower third according to the predominant epicenter of the lesion. CT attenuation values (CTAV) of the tumor parenchyma and abdominal aorta were measured on portal venous phase images, and the normalized tumor enhancement ratio (NTER) was calculated as CTAVtumor/CTAVaorta. Two radiologists independently assessed semantic CT variables while blinded to pathological outcomes; disagreements were resolved by consensus with a senior radiologist.

### CT examination

Complete abdominal CT scans with contrast were administered to all patients no later than one month before surgery. Before scanning, patients were told to drink 800–1000 mL of water over 15–20 minutes. Patients were positioned supine for imaging, which encompassed the area between the diaphragm and the pubic symphysis. The first institution made use of a 64-slice Siemens Somatom Definition AS Silver CT system, while the second employed a 64-slice GE Optima CT660 scanner. Slice intervals of 0.6 to 1.25 mm and thicknesses of 3 to 5 mm were among the scanning parameters, along with an automated tube current modulation and a tube voltage of 120 kV. Images were taken during the portal venous phase, around 65–70 seconds after injection, of intravenous contrast media (320 mg/mL iohexol or 350 mg/mL iopromide) administered at 3.0 mL/s, with a dosage calculated at 1.5 mL/kg.

### ROI segmentation

**Figure 2:**
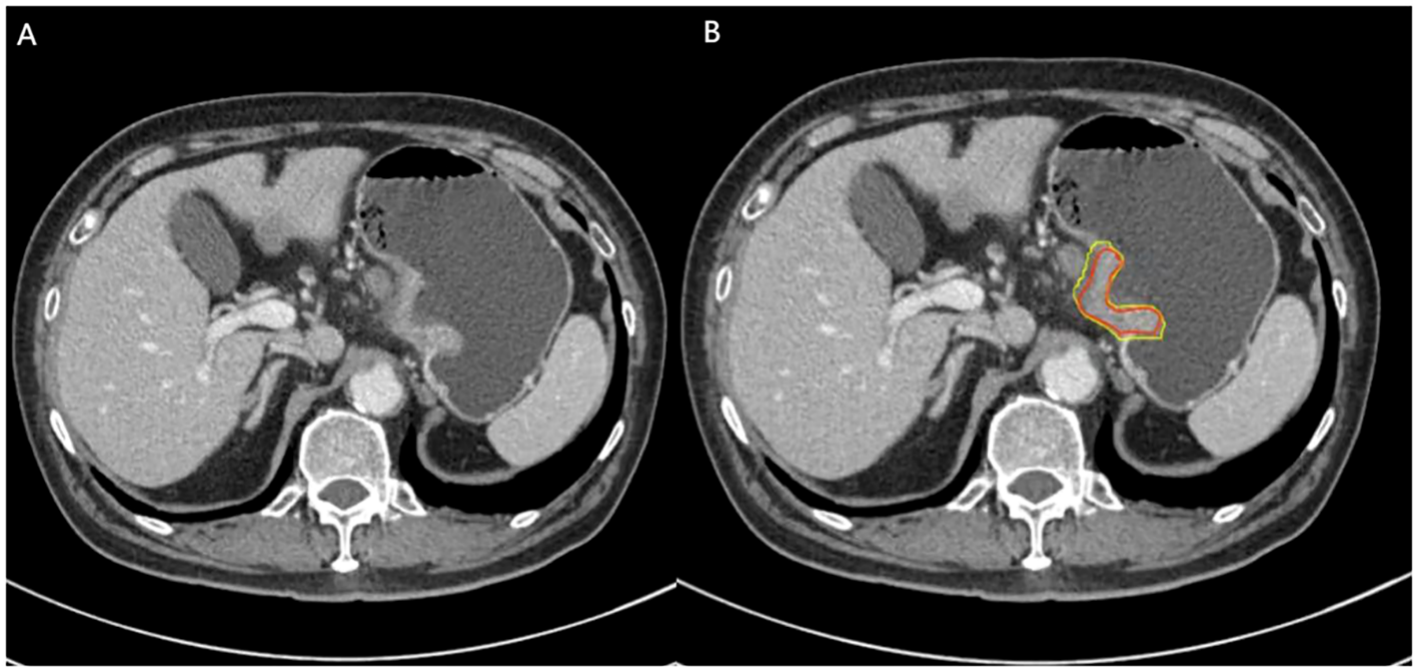
Examples of manual region-of-interest delineation on a portal venous phase CT slice. The intratumoral region (IR) was delineated as VOI1, and the intratumoral plus 3-mm peritumoral region (IPR) was delineated as VOI2 after expansion and manual correction to exclude adjacent bowel, vessels, and organs.

Patient CT imaging data were exported in DICOM (Digital Imaging and Communications in Medicine) format. Venous-phase images were subsequently analyzed using the Dr. Wise Multimodal Research Platform (version 2.7.5), deployed on the Alibaba Cloud platform. Before upload and analysis, DICOM files were anonymized using Washfile to remove sensitive patient identifiers. Two senior radiologists, blinded to clinical and pathological results, independently performed manual delineation of the regions of interest (VOIs) on axial slices. Beginning at the lesion center, continuous voxel-of-interest (VOI) contours were drawn on every consecutive slice, yielding two distinct volumes: VOI1 (encompassing only the tumor core) and VOI2 (containing both the tumor core and peritumoral region). Within the Dr. Wise platform, the built-in “Lesion Expansion” function was applied to isotropically expand VOI1 by 3 mm. The resulting envelope was then manually edited slice-by-slice to exclude bowel loops, major vessels, and adjacent organs, finalizing VOI2. The complete delineation workflow is illustrated in **Figure 2**. Any inter-observer discrepancies regarding tumor boundaries were resolved through consensus. Interobserver and intraobserver reproducibility were assessed in 30 randomly selected cases using intraclass correlation coefficients (ICCs; two-way random-effects, absolute-agreement, single-measure model).

### Radiomics feature extraction and selection

Following automated resampling of venous-phase CT images, radiomics analysis generated 2,153 features from each region of interest using the Dr. Wise software platform. These comprised 414 first-order statistical parameters, 14 shape descriptors, 552 parameters from the gray-level co-occurrence matrix (GLCM), 368 features from the gray-level size-zone matrix (GLSZM), 368 attributes from the gray-level run-length matrix (GLRLM), 322 features from the gray-level dependence matrix (GLDM), and 115 characteristics from the neighborhood gray-tone difference matrix (NGTDM). Feature nomenclature followed standard radiomics feature families described by the Image Biomarker Standardization Initiative (38, 39). Because the platform cannot provide formal documentation certifying IBSI compliance, we report the platform version and extracted feature families for transparency rather than claiming formal IBSI compliance. The machine-learning modules in Dr. Wise use Python-based packages, primarily the scikit-learn library, for model construction and evaluation. Feature standardization parameters were estimated in the training cohort and then applied to the external validation cohort. Reproducible features with interobserver and intraobserver ICCs greater than 0.75 were retained.

**Figure 3:**
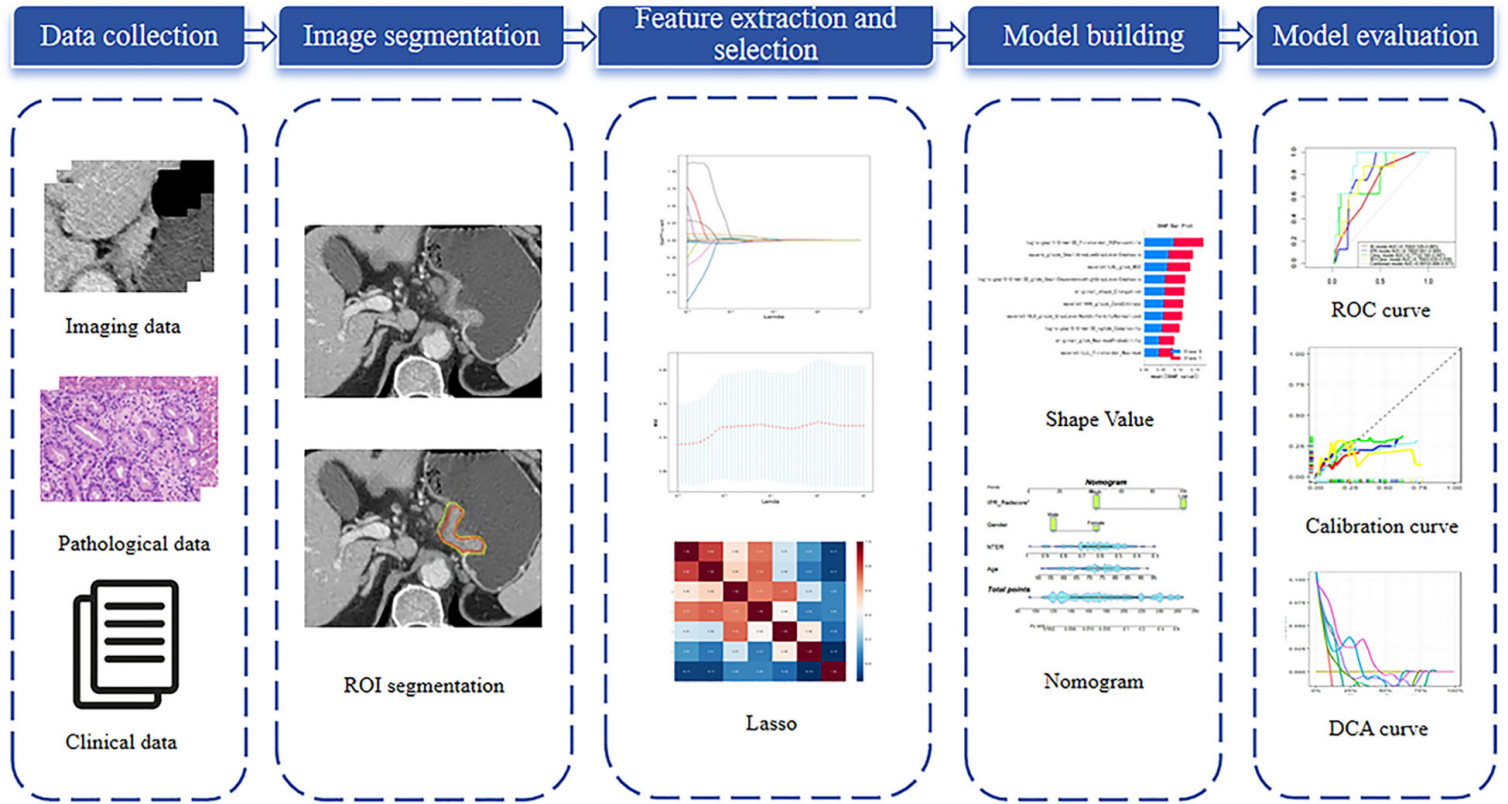
Study workflow. The study design encompassed image generation, tumor segmentation, radiomic feature extraction and selection, modeling, and final evaluation.

Next, correlation analysis was performed to eliminate features with correlation coefficients greater than 0.9. For highly correlated feature pairs in the training set, one redundant feature from each pair was systematically removed. Subsequently, L1-regularized logistic regression was applied for feature selection, generating a sparse coefficient matrix from the training data. The parameter C, controlling the strictness of feature selection, was selected within the training cohort using the Dr. Wise platform’s built-in L1-regularized logistic-regression tuning procedure; lower C values retained fewer features. The screening process is illustrated in **Figure 3**. The resulting continuous IRscore and IPRscore were used to evaluate the radiomics-only models, whereas training-derived cutoffs were used to generate binary IR_Radscore and IPR_Radscore variables for the final clinical-radiomics logistic models. No external-validation labels were used during feature selection, threshold selection, or model fitting.

### Model construction and evaluation

Support-vector machine (SVM) algorithms were used to construct the radiomics-only prediction models based on IR and IPR features. The SVM models were implemented in the Dr. Wise platform as support-vector classifiers (SVC) with probability estimation enabled (probability = true). Hyperparameter tuning was performed in the training cohort over the following grid: C = 0.001, 0.01, 0.1, 1, and 10; kernel = linear or radial basis function (RBF); and max_iter = 50, 100, 150, 200, 300, 500, 800, and 1000. The optimal SVM configuration was selected within the training cohort using the Dr. Wise platform’s built-in tuning procedure, and the external validation cohort was not used during hyperparameter tuning. The gamma parameter for the RBF kernel was not tuned separately and was kept at the default setting of the Dr. Wise/scikit-learn SVC implementation (gamma = ‘scale’). The final selected SVM configuration was C = 0.01, RBF kernel, gamma = ‘scale’, max_iter = 800, and probability = true. The final clinical-radiomics models were built by combining the binary radiomics variables (IR_Radscore or IPR_Radscore) with independent clinical variables in multivariable logistic regression. Univariate analysis identified potential predictors of MSI status among clinical and semantic CT features using a p-value threshold of 0.10. A clinical model was built using stepwise backward elimination guided by the Akaike information criterion. Model comparisons were prespecified as exploratory; the independent external validation cohort was used once for final assessment rather than iterative tuning.

The independent validation cohort was used to evaluate all models. Discrimination was assessed using ROC curves and AUCs. The DeLong test, net reclassification improvement (NRI), and integrated discrimination improvement (IDI) were used for model comparison. No balancing method or class-weighting strategy was applied, including SMOTE, oversampling, undersampling, or class weights. Given the low MSI-H prevalence, we additionally reported MSI-H precision, recall, F1-score, balanced accuracy, Matthews correlation coefficient (MCC), and precision-recall AUC (PR-AUC) for the final combined model. Precision-recall curves and the radiomics/AI reporting checklist were prepared as supplementary files. Calibration was assessed using calibration plots, Brier score, calibration intercept, and calibration slope. Decision-curve analysis (DCA) was used to evaluate net benefit across clinically relevant threshold probabilities.

### MSI status detection

MSI status was determined using IHC staining to assess the expression of mismatch repair (MMR) proteins. Patients were classified as MSI-L/S (positive for all MMR proteins) or MSI-H (negative for at least one MMR protein).

### Statistical analysis

Continuous variables were assessed for normal distribution using the Kolmogorov-Smirnov test. Variables with normal distribution were expressed as mean ± standard deviation (SD) and compared using the t-test, whereas variables with non-normal distributions were presented as medians (interquartile ranges, IQR) and compared with the Mann-Whitney U test. Chi-square or Fisher’s exact tests were applied to categorical data. Variables identified as significant in univariate analyses underwent stepwise multiple logistic regression. Model discrimination, calibration, clinical utility, and class-imbalance-sensitive metrics were reported as described above. All statistical procedures were conducted with R software, and a two-sided p-value less than 0.05 was considered statistically significant.

## Results

### Clinical characteristics

**Supplementary Table 1** summarizes the clinical characteristics of 193 patients enrolled from two institutions. Institution 1 contributed the training cohort (n = 115; MSI-H: 14 cases, MSI-L/S: 101 cases), and Institution 2 contributed the independent external validation cohort (n = 78; MSI-H: 8 cases, MSI-L/S: 70 cases). The MSI-H prevalence was 12.2% in the training cohort and 10.3% in the validation cohort, highlighting the substantial class imbalance. No significant difference in MSI-H prevalence was observed between institutions (p = 0.681).

### Radiomics model development

**Figure 4:**
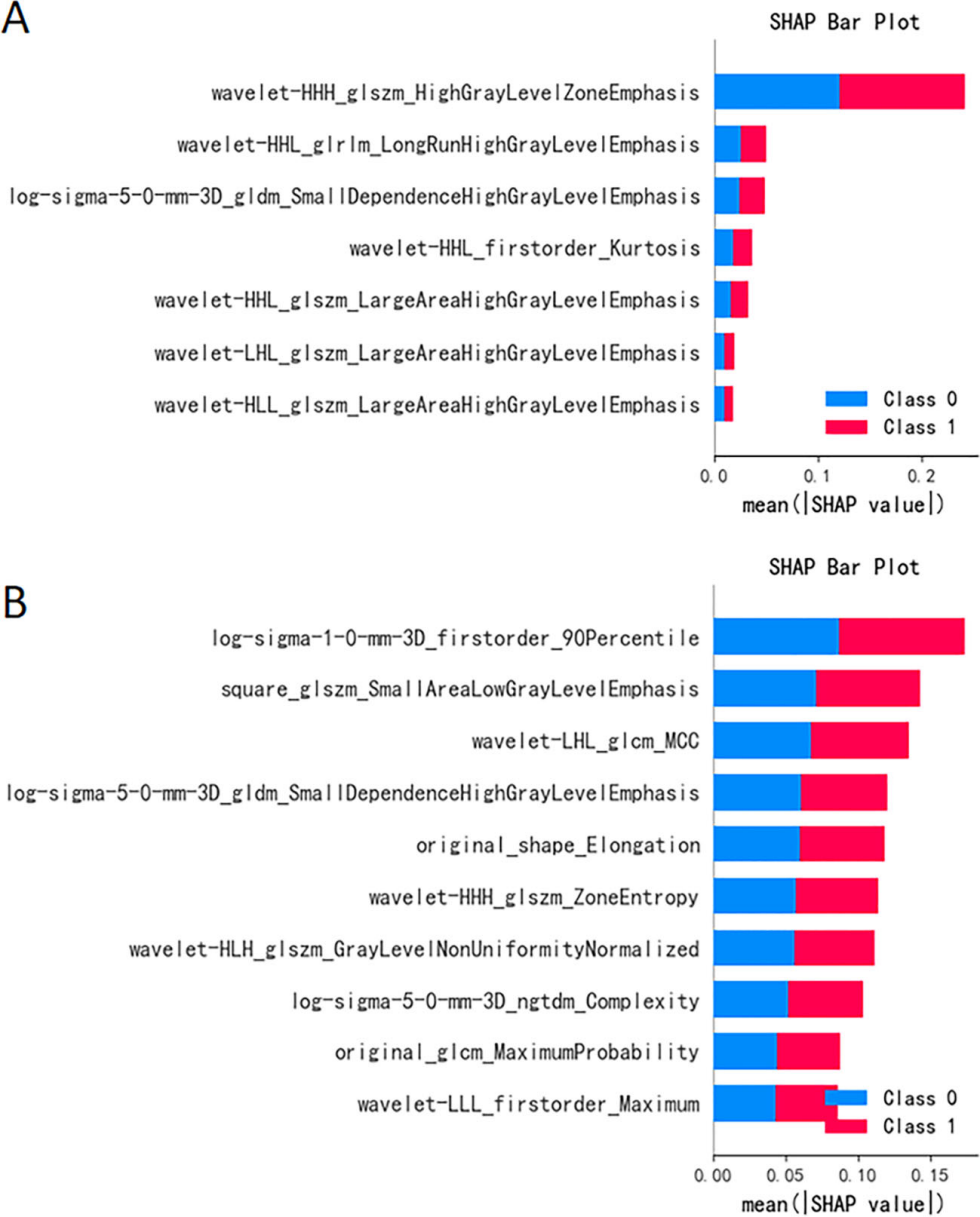
Radiomics features selected in the IR **(A)** and IPR **(B)** models, along with their corresponding coefficients. The x-axis indicates the coefficient values, and the y-axis lists the features.

As shown in **Table 1**, among the two radiomics models employing SVM algorithms, the IPR model exhibited superior predictive capability. The IPR model achieved an AUC of 0.937 (95% CI: 0.868-1.000) in the training set and 0.780 (95% CI: 0.661-0.900) in the validation set. Conversely, the IR model showed a training set AUC of 0.873 (95% CI: 0.761-0.985) but a lower validation set AUC of 0.700 (95% CI: 0.526-0.865). The larger AUC reductions for the IR and IPR radiomics-only models indicate potential overfitting and scanner- or center-related transportability effects, rather than uniformly minimal overfitting. **Figure 4A** shows seven selected IR features, whereas **Figure 4B** shows ten selected IPR features.

**Table 1:** Predictive performance comparison of the two radiomics-only models in the training and external validation cohorts.

Model	Datasets	AUC(95%CI)	Accuracy	Sensitivity	Specificity
IPR(SVM)					
	Training set	0.937(0.868-1)	90.4%	91.2%	84.6%
	Validation set	0.780(0.661-0.900)	78.2%	80.0%	62.5%
IR(SVM)					
	Training set	0.873(0.761-0.985)	83.5%	86.3%	61.5%
	Validation set	0.700(0.526-0.865)	80.8%	87.1%	25.0%

SVM, Support Vector Machine; IR, Intratumoral Region; IPR, Intratumoral plus 3-mm Peritumoral Region; AUC, Area Under the Curve.

### Clinical and combined model construction

Using univariate and multivariate logistic regression analyses, a clinical prediction model incorporating sex, age, and NTER was developed. Multivariate analysis results presented in **Supplementary Table 2** revealed sex (OR = 0.24, 95% CI: 0.06-0.86, p = 0.028), age (OR = 1.10, 95% CI: 1.01-1.20, p = 0.038), and NTER (OR < 0.01, 95% CI: <0.01-0.16, p = 0.012) as statistically significant predictors of MSI status. These variables were integrated with radiomic features to establish two additional models: the IR-plus-clinical (IR + Clinical) and the combined (intratumoral and peritumoral radiomics with clinical characteristics) models.

### Evaluation of model performance

**Figure 5:**
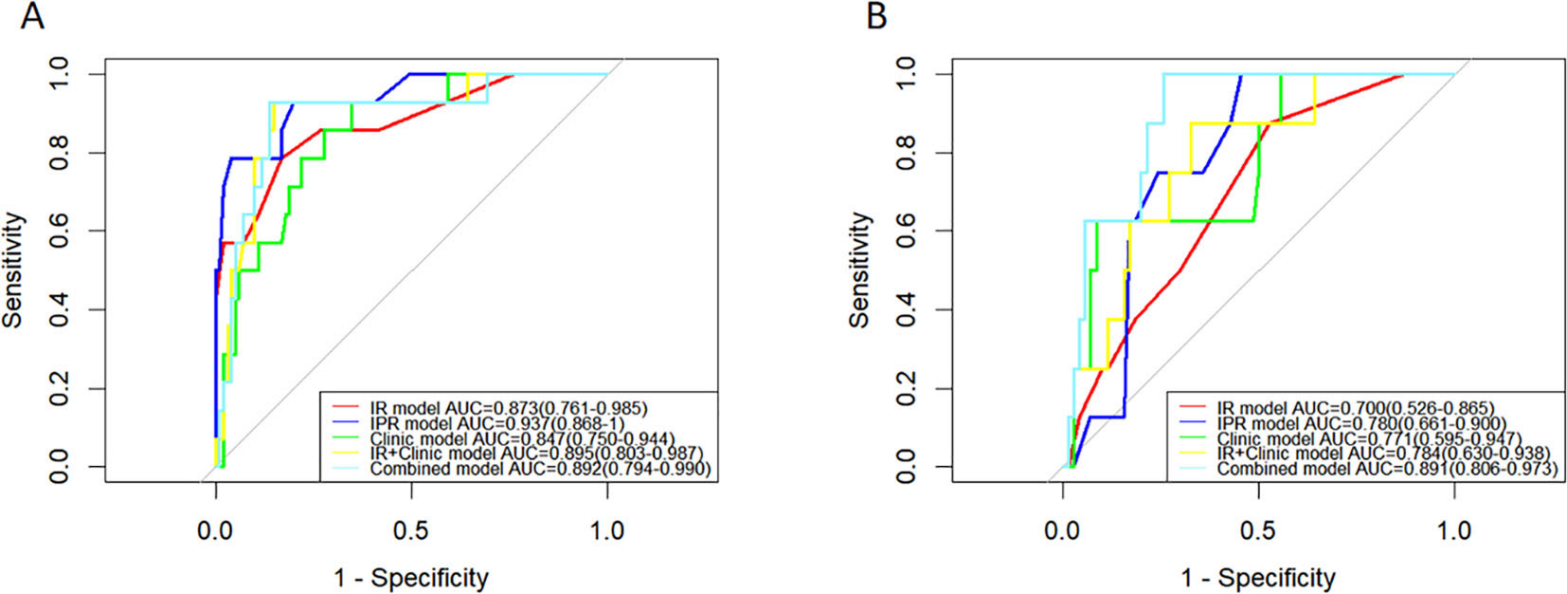
ROC curves of each model for predicting MSI status in the training cohort **(A)** and external validation cohort **(B)**.

As presented in **Table 2** and **Figure 5**, the combined model achieved the highest discriminative performance in the validation cohort (AUC = 0.891; 95% CI: 0.806–0.976), outperforming the IR (AUC = 0.700), IPR (AUC = 0.780), and clinical-only (AUC = 0.771) models. In contrast to the radiomics-only models, the combined model showed similar training and validation AUCs (0.892 and 0.891, respectively), suggesting more stable external validation performance. The final combined model had an accuracy of 82.1%, sensitivity of 0.625, specificity of 0.843, PPV of 0.313, and NPV of 0.952 in the validation cohort.

**Table 2:** Performance metrics of the five prediction models in the training and external validation cohorts.

Model	Datasets	AUC(95%CI)	Accuracy	Sensitivity	Specificity	PPV	NPV
IR(SVM)							
	Training set	0.873(0.761-0.985)	83.5%	86.3%	61.5%	0.364	0.946
	Validation set	0.700(0.526-0.865)	80.8%	87.1%	25.0%	0.182	0.910
IPR(SVM)							
	Training set	0.937(0.868-1)	90.4%	91.2%	84.6%	0.550	0.979
	Validation set	0.780(0.661-0.900)	78.2%	80.0%	62.5%	0.263	0.949
Clinic							
	Training set	0.847(0.750-0.944)	68.7%	92.9%	65.3%	0.271	0.985
	Validation set	0.771(0.595-0.947)	59.0%	62.5%	58.6%	0.147	0.932
IR+Clinic							
	Training set	0.895(0.803-0.987)	86.1%	92.9%	85.1%	0.464	0.989
	Validation set	0.784(0.630-0.938)	80.8%	62.5%	82.9%	0.294	0.951
Combined							
	Training set	0.892(0.794-0.990)	87.0%	92.9%	86.1%	0.481	0.989
	Validation set	0.891(0.806-0.976)	82.1%	62.5%	84.3%	0.313	0.952

SVM, Support Vector Machine; IR, Intratumoral Region; IPR, Intratumoral plus 3-mm Peritumoral Region; AUC, Area Under the Curve; PPV, Positive Predictive Value; NPV, Negative Predictive Value.

As shown in **Table 3**, the combined model achieved an AUC increase of 0.12 compared with the clinical model in the validation cohort (*p* = 0.032). Although comparison with the IPR model did not reach conventional statistical significance (*p* = 0.074), both IDI and NRI were significant (*p* < 0.05), confirming the added value of peritumoral radiomics. While AUC improvement over the IR-plus-clinical model (IR + Clinical) was not statistically significant (*p* = 0.133), the NRI remained significant at 0.36 (*p* = 0.038). This indicates that approximately 36% of patients were correctly reclassified at commonly used risk thresholds, enhancing the practical predictive value of the combined model.

**Table 3:** Comparative predictive performance of the combined model versus IR radiomics, IPR radiomics, clinical, and intratumoral-radiomics-plus-clinical models in the external validation cohort.

Dataset	Model type	Delong (*p*-value)	IDI (95%CI)	*P*-value	NRI (95%CI)	*P*-value
Training set						
	Combined VS. IR	0.688	-0.089 (-0.246-0.069)	0.272	0.103 (-0.175-0.381)	0.467
	Combined VS. IPR	0.099	-0.273 (-0.446--0.099)	0.002**	-0.131 (-0.378-0.116)	0.300
	Combined VS. Clinic	0.067	0.111 (0.080-0.142)	<0.001**	0.236 (-0.007-0.480)	0.057
	Combined VS. IR + Clinic	0.838	-0.009 (-0.078-0.059)	0.789	0.062 (-0.184-0.307)	0.623
Validation set						
	Combined VS. IR	0.056	0.180 (0.008-0.352)	0.041*	0.375 (-0.117-0.867)	0.135
	Combined VS. IPR	0.074	0.191 (0.065-0.317)	0.003**	0.471 (0.118-0.825)	0.009**
	Combined VS. Clinic	0.032*	0.095 (0.062-0.127)	<0.001**	0.096 (-0.143-0.336)	0.429
	Combined VS. IR + Clinic	0.133	0.096 (-0.036-0.227)	0.153	0.361 (0.020-0.702)	0.038*

*p < 0.05, **p < 0.01.

CI, Confidence Interval; IDI, Integrated Discrimination Index; NRI, Net Reclassification Index.

**Figure 6:**
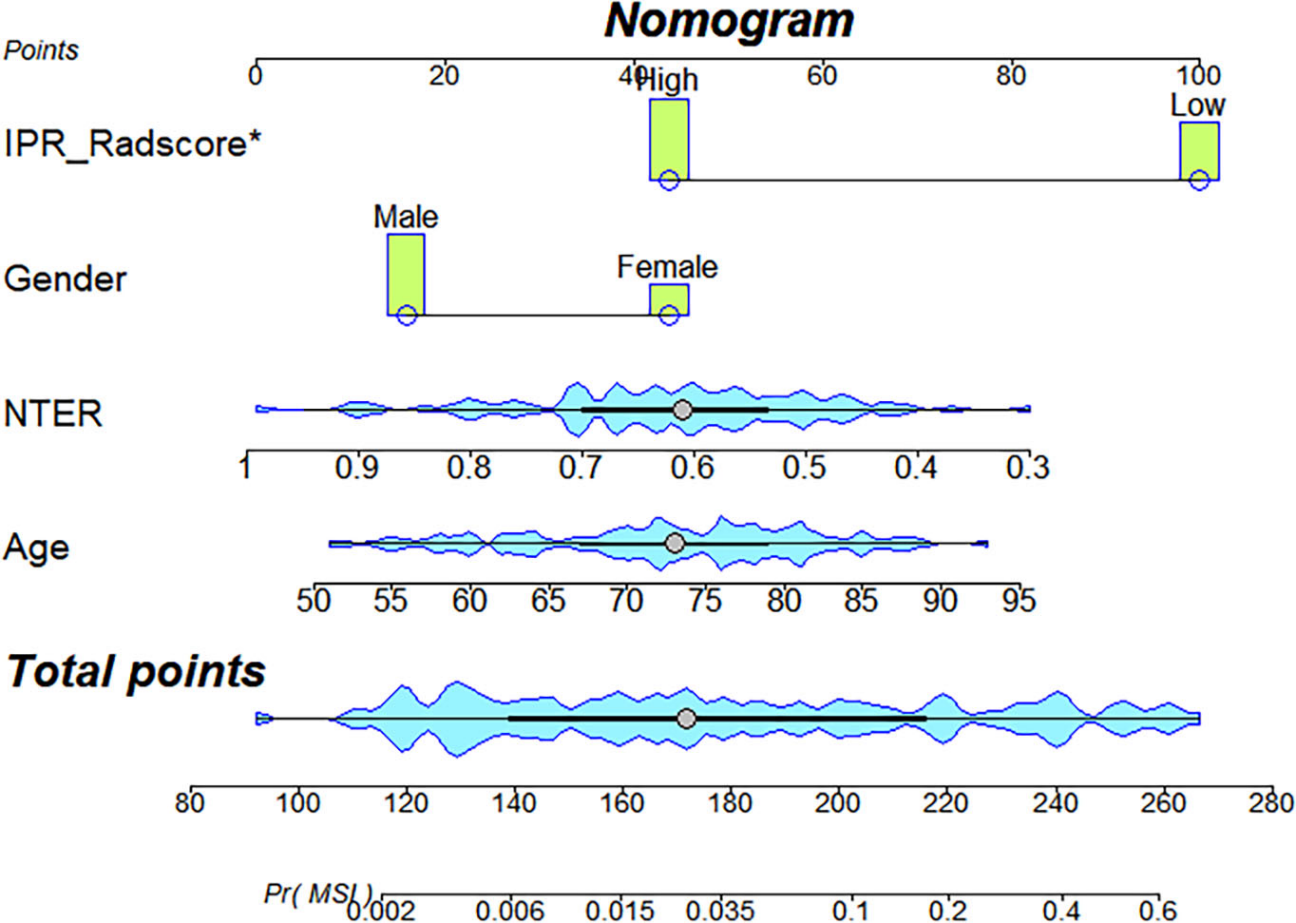
Nomogram of the combined model for predicting MSI-H gastric cancer. For each predictor, draw a vertical line upward to the Points scale to obtain the corresponding score, sum all points, and locate the total on the Total Points scale. A vertical line downward then provides the predicted probability of MSI-H status.

**Figure 7:**
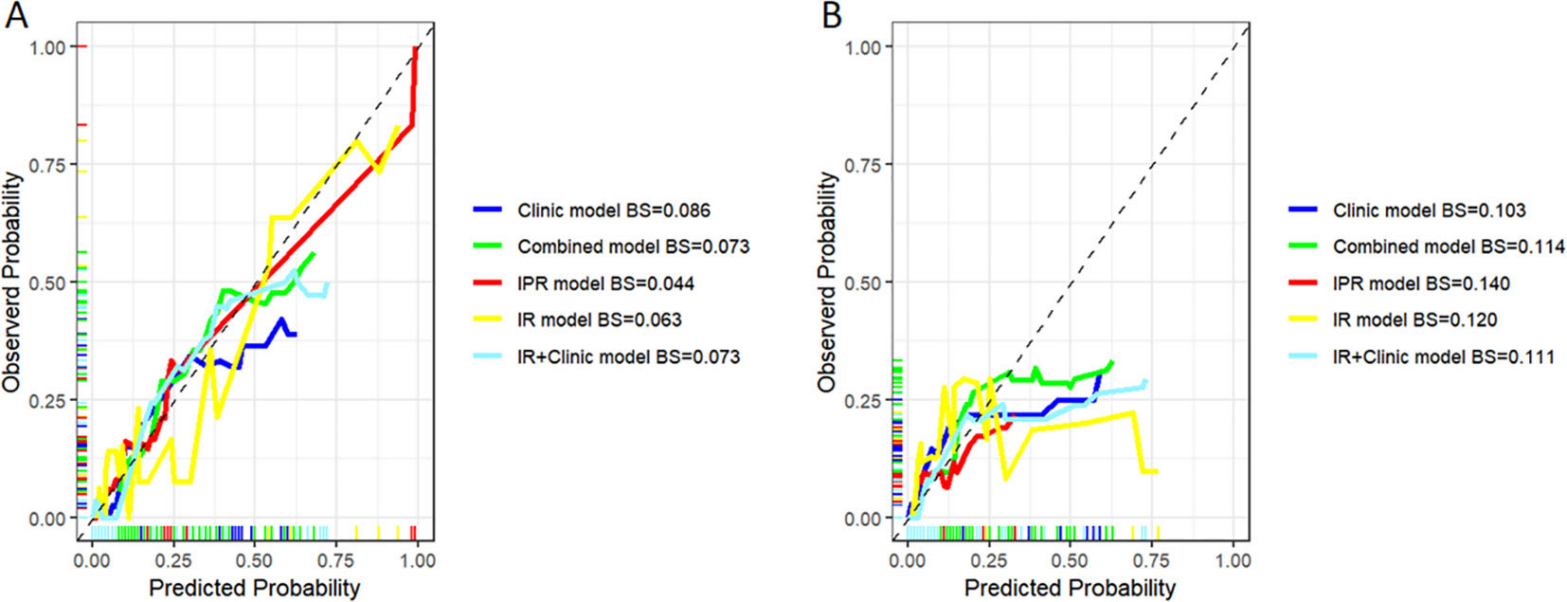
Calibration curves for prediction models in the training cohort **(A)** and external validation cohort **(B)**. BS, Brier Score.

**Figure 8:**
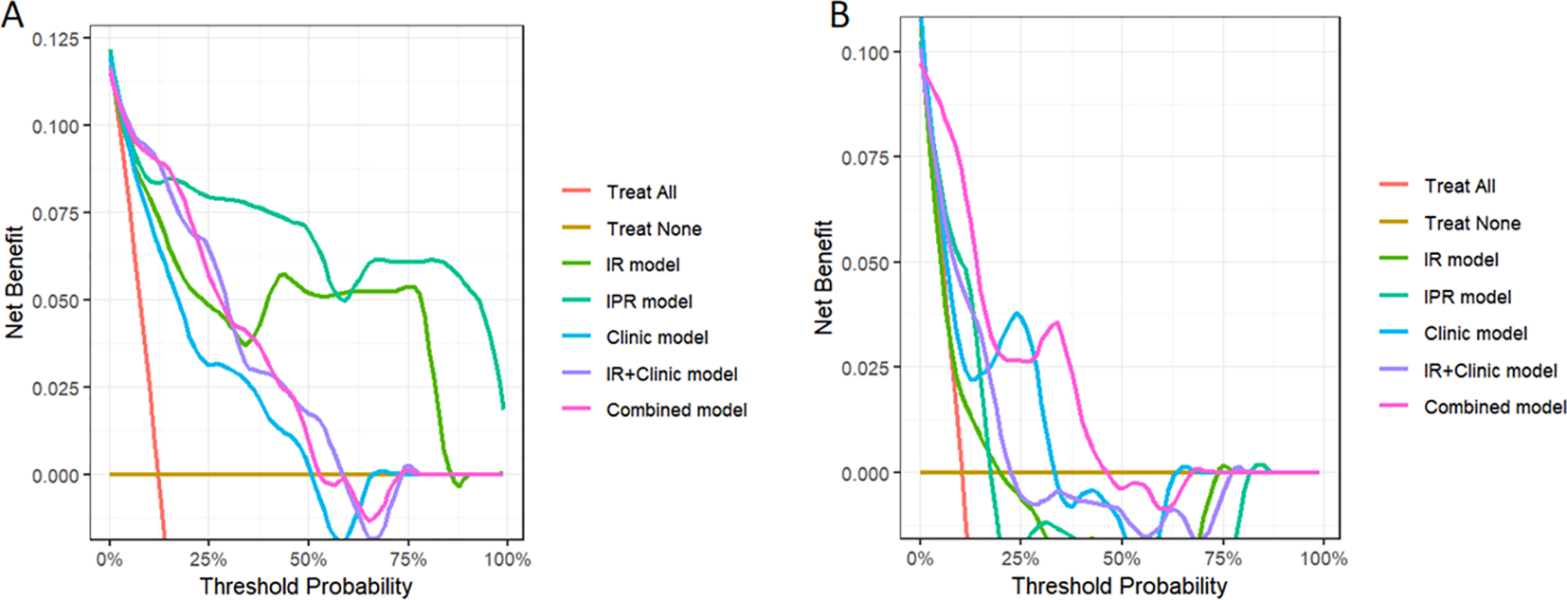
Decision curve analysis (DCA) for each model in the training cohort **(A)** and external validation cohort **(B)**. The x-axis represents threshold probability and the y-axis represents net benefit. A model is considered clinically useful across a threshold range when its net-benefit curve is above both the treat-all and treat-none strategies.

**Figure 6** presents a nomogram of the combined model for individualized MSI-H risk estimation. Calibration curves (**Figure 7**) yielded Brier scores below 0.25, and additional calibration analysis of the final combined model showed a validation-cohort calibration intercept of -0.020 and calibration slope of 1.235. Precision-recall curves were added to address the low MSI-H prevalence (**Supplementary Figure 1**). In the validation cohort, the combined model achieved the highest AUPRC among the five models (AUPRC = 0.454), compared with the clinical model (0.324), IR model (0.233), IPR model (0.240), and IR-plus-clinical model (0.304). Under class imbalance, the combined model also achieved validation-cohort precision of 0.313, recall of 0.625, F1-score of 0.417, balanced accuracy of 0.734, MCC of 0.352, and NPV of 0.952. DCA (**Figure 8**) indicated that the combined model provided greater net benefit than the clinical, radiomics-only, and IR-plus-clinical models over selected threshold probabilities, although this finding requires prospective confirmation.

## Discussion

This dual-center study developed a CT-based clinical-radiomics model that integrated clinical variables with intratumoral and 3-mm peritumoral radiomics features for preoperative MSI prediction in GAC. The combined model achieved an external validation AUC of 0.891, exceeding the clinical-only, IR-only, and IPR-only models. However, because the study was retrospective and included only 22 MSI-H cases, these findings should be interpreted as preliminary evidence that peritumoral radiomics may add information to clinical variables, rather than proof of clinical readiness.

MSI-H tumors are often associated with immune-enriched microenvironments, including CD8+ T-cell and interferon-related immune activity (40). In the present study, IPR features may have captured imaging heterogeneity at the tumor-stromal interface. Nevertheless, no histologic, immunohistochemical, transcriptomic, or spatial-omics validation was performed to directly link specific CT textures with immune infiltration. Therefore, the biological interpretation of the selected radiomics features should be considered hypothesis-generating.

The radiomic features in the IPR model quantified voxel intensity patterns and spatial distributions, reflecting tumor heterogeneity and local tissue interactions (39, 41, 42). In SHAP analysis (**Figure 4B**), log-sigma-1-0-mm-3D_firstorder_90Percentile and square_glszm_SmallAreaLowGrayLevelEmphasis (SALGLE) were identified as key predictors. These features may represent high-intensity and low-gray-level heterogeneity patterns in the peritumoral region, but their biological meaning remains uncertain without independent pathologic or molecular validation.

Sex, age, and NTER were independent clinical predictors; but the clinical-only model showed lower validation performance than the combined model. Adding IPR-derived radiomics improved validation AUC and NPV; however, the PPV remained modest because MSI-H prevalence was low. Thus, the model should not be used as a stand-alone replacement for tissue-based MSI testing. If prospectively validated, it may serve as a non-invasive triage tool to prioritize confirmatory MSI testing, identify patients who may benefit from multidisciplinary review, or support individualized preoperative planning.

This study has several limitations. First, it was retrospective and included a limited number of MSI-H cases (14 in the training cohort and 8 in the external validation cohort), which increases uncertainty, limits multivariable model stability, and contributes to modest PPV despite high NPV. In the training cohort, the final multivariable combined model included four predictors, namely sex, age, NTER, and IPR_Radscore, with 14 MSI-H events, corresponding to approximately 3.5 events per variable. This low events-per-variable value indicates that model stability remains constrained; therefore, the model should be considered exploratory and requires validation in larger MSI-H cohorts. Second, although the validation cohort came from a different institution and CT scanner, the external validation sample was small and may not represent the full heterogeneity of gastric cancer populations. Third, feature selection was not repeated within nested cross-validation; therefore, training-cohort performance may still be optimistic. Fourth, no ComBat or scanner-effect harmonization was applied despite the use of Siemens and GE CT platforms. Fifth, the 3-mm peritumoral margin was selected *a priori*, but sensitivity analyses comparing 1-, 3-, and 5-mm margins were not performed. Sixth, MSI status was determined by IHC rather than PCR or next-generation sequencing, which may introduce reference-standard misclassification. Seventh, only portal venous phase CT images were analyzed; arterial or multiphasic imaging may provide additional information. Eighth, manual segmentation is labor-intensive and may limit routine implementation, so semi-automated or automated segmentation should be evaluated. Finally, the clinical model included age, sex, and NTER but not all potentially relevant clinicopathological variables. Prospective multicenter studies with larger MSI-H samples, harmonized imaging protocols, molecular confirmation, automated segmentation, and prespecified reporting according to radiomics and AI prediction-model guidelines are needed (43–47).

## Conclusion

In summary, integrating CT radiomics from the tumor core and a 3-mm peritumoral region with clinical variables showed promising performance for preoperative MSI prediction in gastric cancer. The model may provide complementary, non-invasive risk information, but it should not replace tissue-based MSI testing. Prospective multicenter validation, larger MSI-H cohorts, multiphasic imaging evaluation, and molecular/pathologic validation are required before this approach can be used to guide clinical decision-making.

## Data Availability

Due to restrictions imposed by the institutional ethics committee and patient privacy regulations, the medical imaging data and clinical information used in this study are not publicly available. The dataset contains identifiable medical images and sensitive clinical records, and public sharing of these data was not covered by the informed consent obtained from the participants. However, de-identified data are available from the corresponding author upon reasonable request and with approval from the institution.
