# Supplementary material for: Interpretable intratumoral and peritumoral radiomics using SHAP for predicting microsatellite status in gastric adenocarcinoma: a dual-center study
Source: Front Oncol. 2026 Jul 20;16:1874404. doi: 10.3389/fonc.2026.1874404 (PMC13429396; doi:10.3389/fonc.2026.1874404)
Supplement: Supplementary file 1 [file DataSheet1.docx]

Supplementary Material

# Supplementary Tables

**Supplementary Table 1.** Demographic and clinical characteristics of the study subjects across both institutions.

| Variables | Total (n = 193) | Institution 1  (n = 115) | Institution 2  (n = 78) | *P* |
| --- | --- | --- | --- | --- |
|  |  |  |  |  |
| Age, Mean ± SD | 71.59 ± 9.11 | 72.69 ± 9.12 | 69.96 ± 8.91 | 0.041* |
| NTER, Mean ± SD | 0.60 ± 0.13 | 0.62 ± 0.12 | 0.58 ± 0.14 | 0.020* |
| BMI, Mean ± SD | 22.28 ± 4.08 | 22.29 ± 4.43 | 22.26 ± 3.52 | 0.956 |
| NLR, M (Q₁, Q₃) | 2.94 (2.28, 3.85) | 2.91 (2.25, 3.87) | 2.95 (2.43, 3.81) | 0.436 |
| Gender, n (%) |  |  |  | 0.416 |
| Female | 48 (24.87) | 31 (26.96) | 17 (21.79) |  |
| male | 145 (75.13) | 84 (73.04) | 61 (78.21) |  |
| Location, n (%) |  |  |  | 0.002** |
| Upper-third | 13 (6.74) | 5 (4.35) | 8 (10.26) |  |
| Middle-third | 48 (24.87) | 20 (17.39) | 28 (35.90) |  |
| Lower-third | 132 (68.39) | 90 (78.26) | 42 (53.85) |  |
| Borrmann, n (%) |  |  |  | 0.166 |
| Ⅰ | 9 (4.66) | 8 (6.96) | 1 (1.28) |  |
| Ⅱ | 47 (24.35) | 31 (26.96) | 16 (20.51) |  |
| Ⅲ | 127 (65.80) | 70 (60.87) | 57 (73.08) |  |
| Ⅳ | 10 (5.18) | 6 (5.22) | 4 (5.13) |  |
| cT stage, n (%) |  |  |  | 0.948 |
| 1-2 | 45 (23.32) | 27 (23.48) | 18 (23.08) |  |
| 3-4 | 148 (76.68) | 88 (76.52) | 60 (76.92) |  |
| cN stage, n (%) |  |  |  | 0.967 |
| 0 | 64 (33.16) | 38 (33.04) | 26 (33.33) |  |
| 1-3 | 129 (66.84) | 77 (66.96) | 52 (66.67) |  |
| cTNM stage, n (%) |  |  |  | 0.353 |
| Ⅰ | 25 (12.95) | 14 (12.17) | 11 (14.10) |  |
| Ⅱ | 59 (30.57) | 38 (33.04) | 21 (26.92) |  |
| Ⅲ | 90 (46.63) | 49 (42.61) | 41 (52.56) |  |
| Ⅳ | 19 (9.84) | 14 (12.17) | 5 (6.41) |  |
| CEA (mg/ml), n (%) |  |  |  | 0.775 |
| <5 | 159 (82.38) | 94 (81.74) | 65 (83.33) |  |
| ≧5 | 34 (17.62) | 21 (18.26) | 13 (16.67) |  |
| CA199 (U/ml), n (%) |  |  |  | 0.467 |
| <37 | 156 (80.83) | 91 (79.13) | 65 (83.33) |  |
| ≧37 | 37 (19.17) | 24 (20.87) | 13 (16.67) |  |
| AFP (ug/L), n (%) |  |  |  | 0.195 |
| <20 | 184 (95.34) | 112 (97.39) | 72 (92.31) |  |
| ≧20 | 9 (4.66) | 3 (2.61) | 6 (7.69) |  |
| CA125 (U/ml), n (%) |  |  |  | 0.882 |
| <35 | 180 (93.26) | 107 (93.04) | 73 (93.59) |  |
| ≧35 | 13 (6.74) | 8 (6.96) | 5 (6.41) |  |
| Hypoproteinemia, n (%) |  |  |  | 0.203 |
| No | 131 (67.88) | 74 (64.35) | 57 (73.08) |  |
| Yes | 62 (32.12) | 41 (35.65) | 21 (26.92) |  |
| Glycosylated hemoglobin (%), n (%) |  |  |  | 0.058 |
| ≦6 | 150 (77.72) | 84 (73.04) | 66 (84.62) |  |
| >6 | 43 (22.28) | 31 (26.96) | 12 (15.38) |  |
| Anaemia, n (%) |  |  |  | 0.129 |
| No | 67 (34.72) | 35 (30.43) | 32 (41.03) |  |
| Yes | 126 (65.28) | 80 (69.57) | 46 (58.97) |  |
| Tumor length (cm), n (%) |  |  |  | 0.872 |
| <5 | 110 (56.99) | 65 (56.52) | 45 (57.69) |  |
| ≧5 | 83 (43.01) | 50 (43.48) | 33 (42.31) |  |
| MSI, n (%) |  |  |  | 0.681 |
| MSI-L/S | 171 (88.60) | 101 (87.83) | 70 (89.74) |  |
| MSI-H | 22 (11.40) | 14 (12.17) | 8 (10.26) |  |

* *p*<0.05, ** *p*<0.01

SD, Standard Deviation; M, Median; Q₁, 1st Quartile; Q₃, 3rd Quartile; TNM, Tumor Node Metastasis; CEA, Carcino Embryonic Antigen; CA199, Cancer Antigen 199; CA125, Cancer Antigen 125; AFP, Alpha Fetoprotein. NTER, Normalized Tumor Enhancement Ratio; NLR, Neutrophil-to-Lymphocyte Ratio; BMI, Body Mass Index; MSI, Microsatellite Instability.

**Supplementary Table 2.** Univariate and multivariate logistic regression analyses of factors influencing MSI status in gastric adenocarcinoma.

| Variables | Univariate analysis | | | Multivariate analysis | | |
| --- | --- | --- | --- | --- | --- | --- |
|  | β | *P* | OR (95%CI) | β | *P* | OR (95%CI) |
| Gender |  |  |  |  |  |  |
| Female |  |  | 1.00 (Reference) |  |  |  |
| Male | -1.51 | 0.011* | 0.22 (0.07 ~ 0.70) | 1.44 | 0.028* | 0.24 (0.06 ~ 0.86) |
| Location |  |  |  |  |  |  |
| Upper-third |  |  | 1.00 (Reference) |  |  |  |
| Middle-third | 14.62 | 0.993 | 2239200.69 (0.00 ~ Inf) |  |  |  |
| Lower-third | 15.79 | 0.993 | 7182890.54 (0.00 ~ Inf) |  |  |  |
| Borrmann |  |  |  |  |  |  |
| Ⅰ |  |  | 1.00 (Reference) |  |  |  |
| Ⅱ | 0.30 | 0.800 | 1.35 (0.13 ~ 13.47) |  |  |  |
| Ⅲ | -0.10 | 0.928 | 0.90 (0.10 ~ 8.32) |  |  |  |
| Ⅳ | -15.62 | 0.992 | 0.00 (0.00 ~ Inf) |  |  |  |
| cT stage |  |  |  |  |  |  |
| 1-2 |  |  | 1.00 (Reference) |  |  |  |
| 3-4 | 0.68 | 0.394 | 1.97 (0.41 ~ 9.43) |  |  |  |
| cN stage |  |  |  |  |  |  |
| 0 |  |  | 1.00 (Reference) |  |  |  |
| 1-3 | -0.48 | 0.408 | 0.62 (0.20 ~ 1.93) |  |  |  |
| cTNM stage |  |  |  |  |  |  |
| Ⅰ |  |  | 1.00 (Reference) |  |  |  |
| Ⅱ | 1.39 | 0.207 | 4.03 (0.46 ~ 35.23) |  |  |  |
| Ⅲ | 0.14 | 0.901 | 1.16 (0.12 ~ 11.26) |  |  |  |
| Ⅳ | -16.00 | 0.993 | 0.00 (0.00 ~ Inf) |  |  |  |
| CEA≧5(mg/ml) | -16.82 | 0.991 | 0.00 (0.00 ~ Inf) |  |  |  |
| CA199≧37 (U/ml) | -1.34 | 0.207 | 0.26 (0.03 ~ 2.10) |  |  |  |
| AFP≧20 (ug/L) | -14.62 | 0.992 | 0.00 (0.00 ~ Inf) |  |  |  |
| CA125≧35 (U/ml) | -15.67 | 0.991 | 0.00 (0.00 ~ Inf) |  |  |  |
| Hypoproteinemia | 1.01 | 0.082 | 2.75 (0.88 ~ 8.57) |  |  |  |
| Glycosylated hemoglobin>6 (%) | 0.47 | 0.434 | 1.60 (0.49 ~ 5.22) |  |  |  |
| Anaemia | 0.53 | 0.439 | 1.70 (0.44 ~ 6.52) |  |  |  |
| Tumor-length≧5 (cm) | 0.30 | 0.600 | 1.35 (0.44 ~ 4.13) |  |  |  |
| Age | 0.09 | 0.023* | 1.09 (1.01 ~ 1.18) | 0.09 | 0.038* | 1.10 (1.01 ~ 1.20) |
| NTER | -9.02 | 0.003** | 0.00 (0.00 ~ 0.05) | -8.26 | 0.012* | 0.00 (0.00 ~ 0.16) |
| BMI | -0.07 | 0.218 | 0.93 (0.84 ~ 1.04) |  |  |  |
| NLR | -0.18 | 0.332 | 0.83 (0.57 ~ 1.21) |  |  |  |

* *p*<0.05, ** *p*<0.01

OR, Odds Ratio; CI, Confidence Interval. TNM, Tumor Node Metastasis; CEA, Carcino Embryonic Antigen; CA199, Cancer Antigen 199; CA125, Cancer Antigen 125; AFP, Alpha-Fetal Protein. NTER, Normalized Tumor Enhancement Ratio; NLR, Neutrophil-to-Lymphocyte Ratio; BMI, Body Mass Index.

**Supplementary Table 3.** Univariate and multivariate logistic regression were used within the combined model to identify factors influencing microsatellite instability (MSI) status in gastric adenocarcinoma.

| Variables | Univariate analysis | | | Multivariate analysis | | |
| --- | --- | --- | --- | --- | --- | --- |
|  | β | *P* | OR (95%CI) | β | *P* | OR (95%CI) |
| Gender |  |  |  |  |  |  |
| Female |  |  | 1.00 (Reference) |  |  | 1.00 (Reference) |
| Male | -1.51 | 0.011* | 0.22 (0.07 ~ 0.70) | -1.29 | 0.066 | 0.28 (0.07 ~ 1.09) |
| Location |  |  |  |  |  |  |
| Upper-third |  |  | 1.00 (Reference) |  |  |  |
| Middle-third | 14.62 | 0.993 | 2239200.69 (0.00 ~ Inf) |  |  |  |
| Lower-third | 15.79 | 0.993 | 7182890.54 (0.00 ~ Inf) |  |  |  |
| Borrmann |  |  |  |  |  |  |
| Ⅰ |  |  | 1.00 (Reference) |  |  |  |
| Ⅱ | 0.30 | 0.800 | 1.35 (0.13 ~ 13.47) |  |  |  |
| Ⅲ | -0.10 | 0.928 | 0.90 (0.10 ~ 8.32) |  |  |  |
| Ⅳ | -15.62 | 0.992 | 0.00 (0.00 ~ Inf) |  |  |  |
| cT stage |  |  |  |  |  |  |
| 1-2 |  |  | 1.00 (Reference) |  |  |  |
| 3-4 | 0.68 | 0.394 | 1.97 (0.41 ~ 9.43) |  |  |  |
| cN stage |  |  |  |  |  |  |
| 0 |  |  | 1.00 (Reference) |  |  |  |
| 1-3 | -0.48 | 0.408 | 0.62 (0.20 ~ 1.93) |  |  |  |
| cTNM stage |  |  |  |  |  |  |
| Ⅰ |  |  | 1.00 (Reference) |  |  |  |
| Ⅱ | 1.39 | 0.207 | 4.03 (0.46 ~ 35.23) |  |  |  |
| Ⅲ | 0.14 | 0.901 | 1.16 (0.12 ~ 11.26) |  |  |  |
| Ⅳ | -16.00 | 0.993 | 0.00 (0.00 ~ Inf) |  |  |  |
| CEA≧5(mg/ml) | -16.82 | 0.991 | 0.00 (0.00 ~ Inf) |  |  |  |
| CA199≧37 (U/ml) | -1.34 | 0.207 | 0.26 (0.03 ~ 2.10) |  |  |  |
| AFP≧20 (ug/L) | -14.62 | 0.992 | 0.00 (0.00 ~ Inf) |  |  |  |
| CA125≧35 (U/ml) | -15.67 | 0.991 | 0.00 (0.00 ~ Inf) |  |  |  |
| Hypoproteinemia | 1.01 | 0.082 | 2.75 (0.88 ~ 8.57) |  |  |  |
| Glycosylated hemoglobin>6 (%) | 0.47 | 0.434 | 1.60 (0.49 ~ 5.22) |  |  |  |
| Anaemia | 0.53 | 0.439 | 1.70 (0.44 ~ 6.52) |  |  |  |
| Tumor-length≧5 (cm) | 0.30 | 0.600 | 1.35 (0.44 ~ 4.13) |  |  |  |
| IPRscore median |  |  |  |  |  |  |
| 1 |  |  | 1.00 (Reference) |  |  | 1.00 (Reference) |
| 2 | -3.20 | 0.003** | 0.04 (0.01 ~ 0.32) | -2.60 | 0.018* | 0.07 (0.01 ~ 0.64) |
| Age | 0.09 | 0.023* | 1.09 (1.01 ~ 1.18) | 0.08 | 0.086 | 1.08 (0.99 ~ 1.18) |
| NTER | -9.02 | 0.003** | 0.00 (0.00 ~ 0.05) | -5.48 | 0.080 | 0.00 (0.00 ~ 1.94) |
| BMI | -0.07 | 0.218 | 0.93 (0.84 ~ 1.04) |  |  |  |
| NLR | -0.18 | 0.332 | 0.83 (0.57 ~ 1.21) |  |  |  |

* *p*<0.05, ** *p*<0.01

OR, Odds Ratio; CI, Confidence Interval. TNM, Tumor Node Metastasis; CEA, Carcino Embryonic Antigen; CA199, Cancer Antigen 199; CA125, Cancer Antigen 125; AFP, Alpha-Fetal Protein. NTER, Normalized Tumor Enhancement Ratio; NLR, Neutrophil-to-Lymphocyte Ratio; BMI, Body Mass Index.

**Supplementary Table 4.** Additional class-imbalance-sensitive metrics for the final combined model.

| Dataset | PR-AUC | Precision | Recall | F1-score | Balanced  accuracy | MCC | Calibration  intercept | Calibration  slope |
| --- | --- | --- | --- | --- | --- | --- | --- | --- |
| Training cohort | 0.518 | 0.481 | 0.929 | 0.634 | 0.895 | 0.609 | 0.043 | 1.092 |
| Validation set | 0.454 | 0.313 | 0.625 | 0.417 | 0.734 | 0.352 | -0.020 | 1.235 |

PR-AUC, Precision-recall area under the curve; MCC, Matthews correlation coefficient.

**Supplementary Table 5.** Radiomics/AI reporting checklist.

| **PRESENT** | **PARTIAL** | **MISSING** | **NA** |
| --- | --- | --- | --- |
| **37** | **4** | **0** | **1** |

| **No** | **Domain** | **Reporting item** | **Status** | **Location** | **Remarks / action** |
| --- | --- | --- | --- | --- | --- |
| 1 | CLAIM / Title | Title identifies the study as an imaging/radiomics machine-learning study and indicates the clinical task. | PRESENT | Title and Abstract | Retained in the revised manuscript. |
| 2 | CLAIM / Abstract | Abstract reports study design, cohorts, model types, validation, and key performance. | PRESENT | Abstract | Ensure final abstract metrics match the revised Results. |
| 3 | CLAIM / Introduction | Clinical rationale, knowledge gap, and objective are explicitly stated. | PRESENT | Introduction | No further action required. |
| 4 | CLAIM / Intended use | The intended clinical role of the model is described without overclaiming. | PRESENT | Introduction; Discussion | The revised text frames the model as an adjunctive risk-stratification tool. |
| 5 | CLAIM / Study design | Retrospective design, participating centers/cohorts, and validation strategy are reported. | PRESENT | Materials and Methods | Training and external validation cohorts are identified. |
| 6 | CLAIM / Ethics | Ethics approval and consent/waiver are described. | PRESENT | Ethics statement; Materials and Methods | Confirm that approval numbers are unchanged in the final submission system. |
| 7 | CLAIM / Participants | Eligibility criteria, inclusion/exclusion flow, and patient-level cohort construction are reported. | PRESENT | Materials and Methods; Figure 1 | Patient-level partitioning should remain explicit. |
| 8 | CLAIM / Data split | Training and external validation were separated at the patient level, with no test-set tuning. | PRESENT | Materials and Methods | Feature selection and model fitting were restricted to the training cohort. |
| 9 | CLAIM / De-identification | De-identification method is reported. | PRESENT | Materials and Methods | DICOM data were anonymized with Washfile before upload/analysis. |
| 10 | CLAIM / Imaging data | CT phase, acquisition/reconstruction information, and image source are described. | PRESENT | Materials and Methods | Keep scanner/protocol details in the final Methods or supplement. |
| 11 | CLAIM / Reference standard | Outcome label and reference standard for MSI status are described. | PRESENT | Materials and Methods | Maintain blinding/timing details where available. |
| 12 | CLAIM / Annotation | ROI/VOI segmentation method, readers, and reproducibility assessment are reported. | PRESENT | Materials and Methods | ICC threshold greater than 0.75 is reported. |
| 13 | CLAIM / Software | AI/radiomics software platform and version are reported. | PRESENT | Materials and Methods | Dr. Wise Multimodal Research Platform (version 2.7.5) is stated. |
| 14 | CLAIM / Platform deployment | Computing/deployment environment is described sufficiently for reproducibility. | PRESENT | Materials and Methods | Platform deployment on Alibaba Cloud is stated. |
| 15 | CLAIM / ML library | Core machine-learning implementation/library is identified. | PRESENT | Materials and Methods | Python-based modules, primarily scikit-learn, are stated. |
| 16 | CLAIM / Hyperparameters | Model type, tuning grid, and final selected hyperparameters are reported. | PRESENT | Materials and Methods | SVC grid and final C=0.01, RBF kernel, max_iter=800, probability=true, gamma = 'scale' are reported. |
| 17 | CLAIM / Class imbalance | Class prevalence and imbalance-handling strategy are reported. | PRESENT | Materials and Methods; Results | No balancing method or class-weighting strategy was applied. |
| 18 | CLAIM / Evaluation metrics | Discrimination, calibration, clinical utility, and prevalence-sensitive metrics are reported. | PRESENT | Materials and Methods; Results | ROC/AUC, calibration, DCA, PR-AUC, precision, recall, F1, balanced accuracy, and MCC are included. |
| 19 | CLAIM / Uncertainty | Confidence intervals and statistical comparison methods are reported. | PRESENT | Materials and Methods; Results | AUC comparisons use DeLong; retain CIs in final tables. |
| 20 | CLAIM / Limitations | Limitations and generalizability are discussed. | PRESENT | Discussion | Low PPV and retrospective design are acknowledged. |
| 21 | CLEAR / Radiomics workflow | Radiomics pipeline from image selection to feature extraction, selection, modeling, and validation is described. | PRESENT | Materials and Methods | Workflow has been expanded in the revision. |
| 22 | CLEAR / Image preprocessing | Resampling and feature standardization are described. | PRESENT | Materials and Methods | Feature standardization was fit in the training cohort and applied to external validation. |
| 23 | CLEAR / Feature classes | Extracted radiomics feature families and counts are reported. | PRESENT | Materials and Methods | First-order, shape, GLCM, GLSZM, GLRLM, GLDM, and NGTDM counts are listed. |
| 24 | CLEAR / IBSI transparency | IBSI-related terminology and platform limitations are transparently stated. | PARTIAL | Materials and Methods; Response letter | Feature nomenclature follows standard feature families, but the platform cannot provide formal IBSI compliance certification. The manuscript does not claim formal IBSI compliance. |
| 25 | CLEAR / Feature reproducibility | Interobserver and intraobserver reproducibility filtering is reported. | PRESENT | Materials and Methods | Features with ICCs greater than 0.75 were retained. |
| 26 | CLEAR / Harmonization | Scanner/protocol harmonization strategy is reported if applied. | PARTIAL | Materials and Methods | No separate harmonization method is claimed in the current revision. If none was used, keep this transparent. |
| 27 | CLEAR / Feature selection | Feature selection is performed within the training cohort to reduce leakage risk. | PRESENT | Materials and Methods | Training-only feature-selection/model-fitting pipeline is stated. |
| 28 | CLEAR / Model components | Radiomics-only, clinical-only, and combined models are clearly distinguished. | PRESENT | Materials and Methods; Results | Final clinical-radiomics model uses binary IR_Radscore/IPR_Radscore variables. |
| 29 | CLEAR / External validation | Independent validation cohort is reported. | PRESENT | Materials and Methods; Results | Pingyang is the training cohort; Yinzhou is the external validation cohort. |
| 30 | CLEAR / Open science | Data/code/model availability is stated. | PARTIAL | Data Availability; Response letter | Data availability should match institutional/privacy restrictions; platform code is not claimed as open source. |
| 31 | TRIPOD+AI / Model objective | Prediction target, intended users, and intended clinical setting are described. | PRESENT | Introduction; Methods | Endpoint is MSI status prediction/risk stratification. |
| 32 | TRIPOD+AI / Predictors | Candidate predictors and final model predictors are defined. | PRESENT | Materials and Methods; Results | Age, sex, NTER, IR_Radscore, and IPR_Radscore are reported. |
| 33 | TRIPOD+AI / Sample size | Cohort size and number of events are reported. | PRESENT | Results | Training n=115 with 14 MSI-H; validation n=78 with 8 MSI-H. |
| 34 | TRIPOD+AI / Missing data | Missing-data handling is reported. | PARTIAL | Materials and Methods | Confirm that final manuscript states exclusion or imputation procedures explicitly. |
| 35 | TRIPOD+AI / Predictor handling | Continuous/binary predictor handling and transformations are described. | PRESENT | Materials and Methods | Final model used binary radiomics variables; continuous scores were used for radiomics-only PR/ROC analyses. |
| 36 | TRIPOD+AI / Model fitting | Model fitting procedure and hyperparameter tuning are reported. | PRESENT | Materials and Methods | SVC tuning grid and selected parameters are reported. |
| 37 | TRIPOD+AI / Validation | External validation performance is separately reported. | PRESENT | Results | Validation cohort performance is reported separately from training. |
| 38 | TRIPOD+AI / Calibration | Calibration assessment is reported. | PRESENT | Materials and Methods; Results | Calibration plots, Brier score, calibration intercept, and slope are included. |
| 39 | TRIPOD+AI / Clinical utility | Decision-curve analysis is reported. | PRESENT | Materials and Methods; Results | DCA evaluates net benefit across threshold probabilities. |
| 40 | TRIPOD+AI / Threshold performance | Threshold-dependent performance is reported with imbalance-sensitive metrics. | PRESENT | Results | Precision, recall, F1-score, balanced accuracy, MCC, PPV, and NPV are reported. |
| 41 | TRIPOD+AI / Fairness/subgroups | Subgroup or fairness analyses are reported if planned and powered. | NA | Discussion | Not applicable/not performed because cohort size and MSI-H event count were limited; acknowledge as a limitation if challenged. |
| 42 | TRIPOD+AI / Supplementary files | Additional materials supporting reproducibility are uploaded. | PRESENT | Supplementary files | PR curves and this reporting checklist are provided as supplementary files. |

Scope: This checklist summarizes reporting completeness for the revised manuscript. It is not an official IBSI compliance certificate and does not claim formal IBSI compliance.

Status definitions: PRESENT = reported in the revised manuscript; PARTIAL = reported with an explicit limitation or requiring final author confirmation; NA = not applicable to the present study.

Abbreviations: CLAIM = Checklist for Artificial Intelligence in Medical Imaging; CLEAR = CheckList for EvaluAtion of Radiomics research; IBSI = Image Biomarker Standardisation Initiative; NA = not applicable; TRIPOD+AI = Transparent Reporting of a multivariable prediction model for Individual Prognosis Or Diagnosis plus Artificial Intelligence.

**Supplementary Figure 1.**


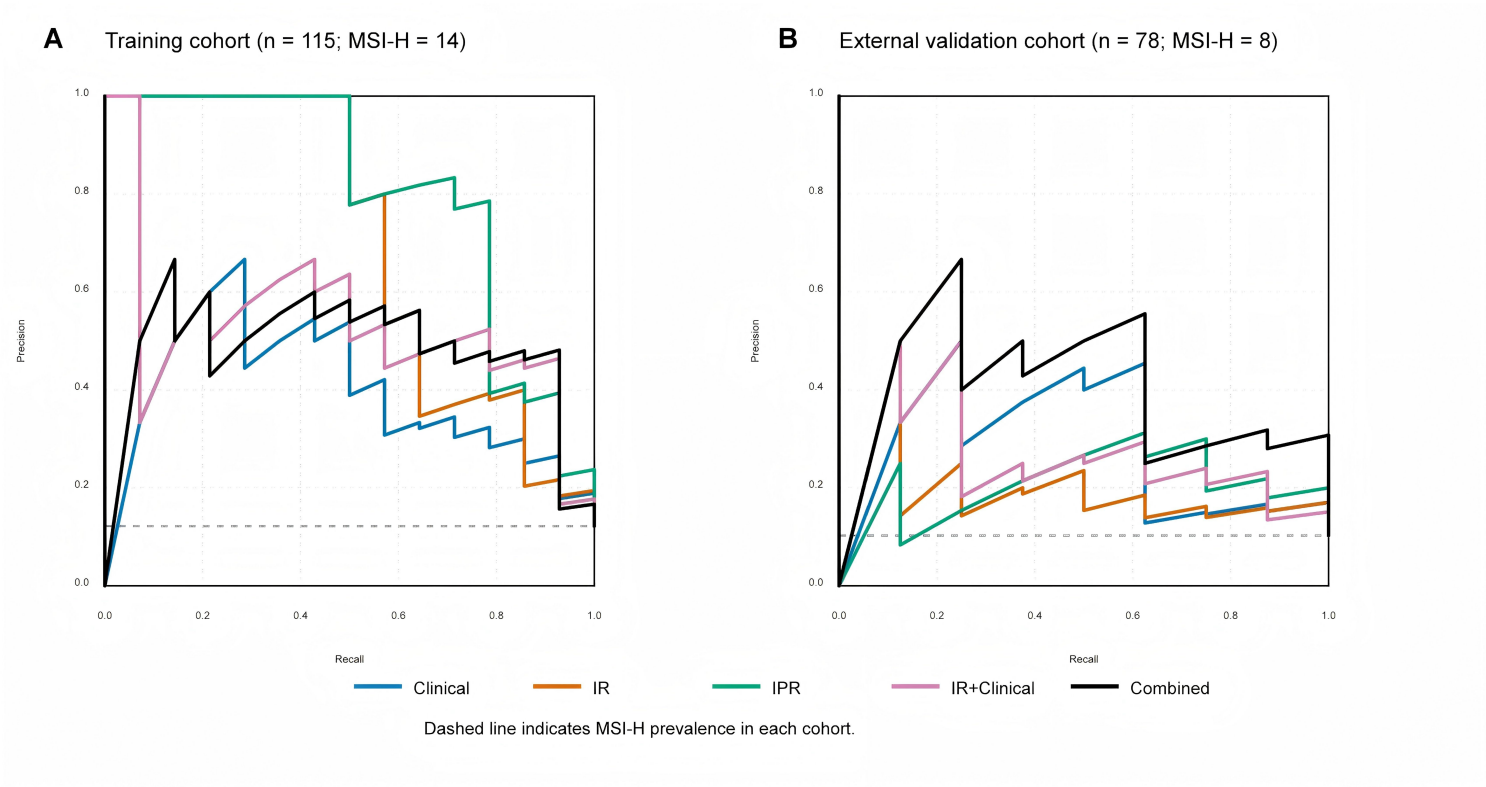


**Supplementary Figure 1.** Precision-recall curves of the clinical, IR radiomics, IPR radiomics, IR-plus-clinical, and combined models in the training cohort (A) and external validation cohort (B). The dashed horizontal line indicates the MSI-H prevalence in each cohort. In the external validation cohort, the combined model showed the highest AUPRC (0.454), supporting its better minority-class ranking performance under class imbalance.
